# The Neural Basis of Visual Search in Scene Context

**DOI:** 10.1177/09637214241304349

**Published:** 2025-02-19

**Authors:** Marius V. Peelen

**Affiliations:** Donders Institute for Brain, Cognition, and Behaviour, Radboud University

**Keywords:** visual search, attentional template, fMRI, visual cortex, prediction

## Abstract

Humans are highly efficient in finding objects in their structured, daily-life environments. Behavioral studies have demonstrated that this efficiency is supported by expectations derived from scene context. Here, I review neuroimaging studies that have started to reveal the neural basis of contextual influences on visual search for objects. These studies point to a central role for the object-selective visual cortex (OSC) in mediating multiple types of contextual influences. Supporting the attentional guidance by scene context, activity patterns in the OSC reflect global contextual expectations about target location and represent local nontarget objects that are contextually associated with the target. Preparatory activity patterns in the OSC also incorporate contextual expectations about target appearance (e.g., object size) during the preparation phase of visual search. In addition to supporting attentional guidance, object representations in the OSC are directly facilitated by scene context, and this facilitation is causally linked to object-identification performance. Finally, activity patterns in the anterior OSC integrate representations of distractor objects that are positioned according to familiar configurations, thereby reducing scene complexity. Together, these studies show how attention and expectation interactively drive preparatory activity and jointly modulate the visual processing of potential targets, providing a neural basis for the efficiency of search in scenes.

Throughout the day, we perform goal-directed tasks that involve visually selecting objects for further processing, such as picking the right spice jar when preparing a dish or finding a product in the supermarket. Through extensive experience in performing such tasks, we have become highly efficient in orienting our attention to familiar objects in cluttered daily-life environments, although we also still make (potentially costly) mistakes. A core aim of visual cognitive neuroscience has been to understand the neural mechanisms underlying the efficient attentional selection of objects in natural scenes.

Much of what is known about the neural basis of attentional selection has come from experiments using simplified stimulus displays. In these experiments, participants are cued to search for a specific target stimulus (e.g., a red cross) within displays consisting of randomly arranged distractor stimuli (e.g., blue crosses and red circles). Using this approach, neurophysiological work in animals has reported that neurons in the visual cortex tuned to target features show sustained activity in the preparation phase of a search trial (i.e., after the cue but before the onset of the stimulus display; Desimone & Duncan, 1995). This preparatory activity acts as a top-down attentional bias and may constitute a neural correlate of the search (or attentional) template (Battistoni et al., 2017; Desimone & Duncan, 1995; Eimer, 2014). Subsequently, when the search display appears, neural processing is biased in favor of items containing target features, thereby guiding spatial attention (and eye movements) toward these candidate targets (Bichot et al., 2005).

An important question is whether and how these results from simplified search tasks generalize to search in more naturalistic settings. Behavioral studies have shown that visual search in (pictures of) natural scenes is much more efficient than visual search in artificial arrays. For example, the time to report the presence of target objects in a natural scene only weakly scales with the number of distractor objects, unlike search in artificial arrays (Neider & Zelinsky, 2008; Wolfe et al., 2011). The remarkable efficiency of naturalistic visual search suggests that its neural basis may differ from the neural basis of artificial visual search (Peelen & Kastner, 2014).

A key feature of naturalistic visual search is the presence of context: the familiar, meaningful, and structured environments (i.e., scenes) within which goal-directed tasks are typically completed. Context provides probabilistic information about the presence of objects (semantic information) and their locations (syntactic information), both within the global scene and relative to other objects (Biederman et al., 1982; for a review, see Võ et al., 2019). Behaviorally, the presence of scene context strongly facilitates visual search performance (for reviews, see Castelhano & Krzyś, 2020; Oliva & Torralba, 2007; Võ et al., 2019). Here, I review neuroimaging work that has started to shed light on the neural basis of contextual influences on naturalistic visual search.

## Global and Local Contextual Guidance in the Visual Cortex

**Fig. 1:**
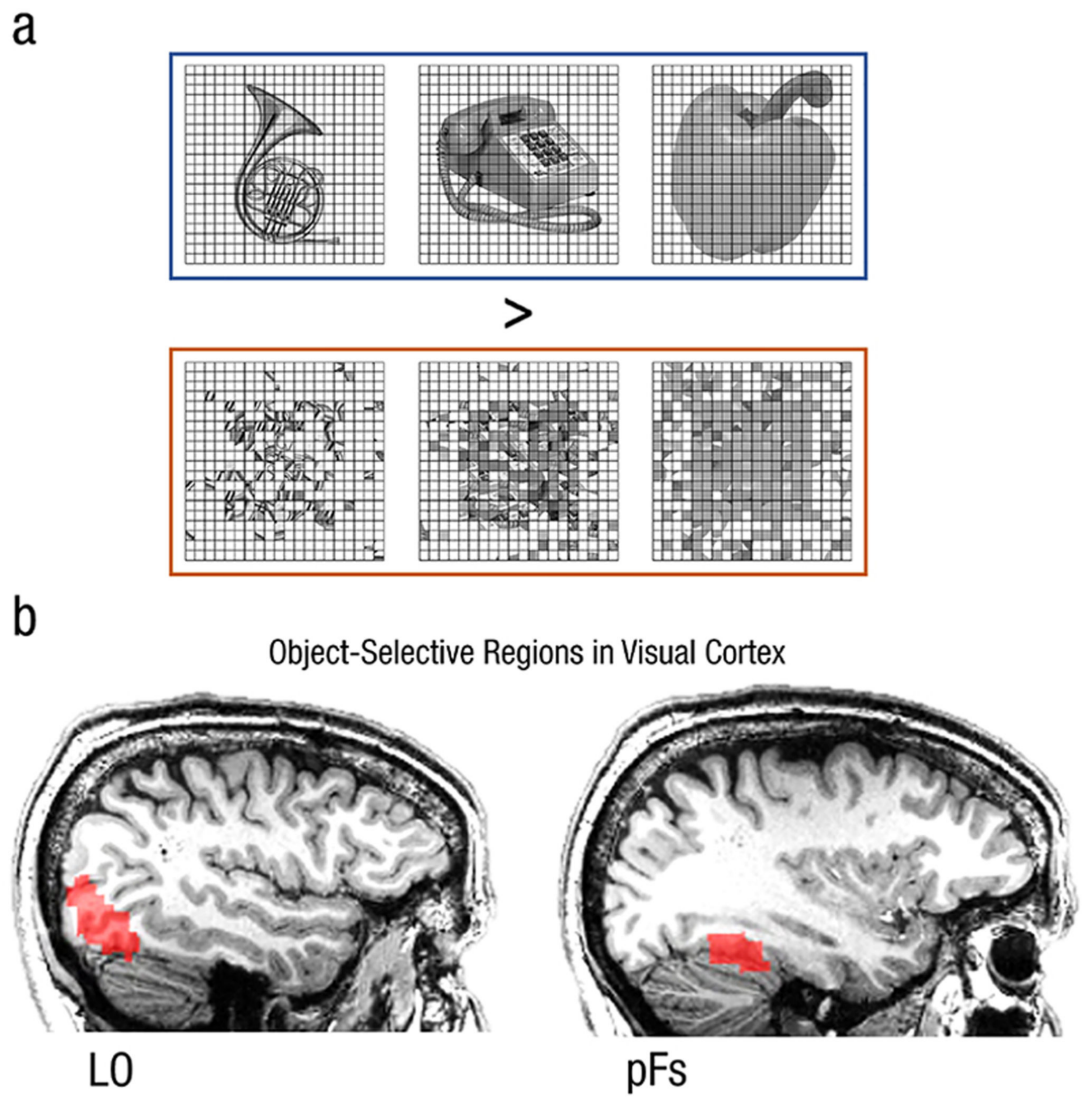
Object-selective regions in the visual cortex. The OSC can be functionally localized by contrasting fMRI responses to (a) intact versus scrambled objects. Object-selective regions, sometimes collectively referred to as the lateral occipital complex or LOC (Grill-Spector et al., 2001), can be found in (b) the lateral occipital (LO) cortex and the posterior fusiform (pFs) gyrus. LO and pFs can be further subdivided using visual field mapping. In the current article, these regions are collectively referred to as the OSC, although the effects described here may be specific to regions within the OSC. The OSC contains representations of objects and object parts and receives both feedforward (from the early visual cortex) and feedback input (e.g., related to top-down attention and expectation). The OSC can be distinguished from the functionally localized scene-selective cortex (defined by the contrast scenes > objects) and face-selective cortex (defined by the contrast faces > objects), although these regions may overlap the OSC when functionally defined at more lenient statistical thresholds. OSC = object-selective visual cortex; fMRI = functional MRI; LO = lateral occipital; pFs = posterior fusiform.

One way in which scene context facilitates search is by guiding spatial attention to possible target locations (for reviews, see Castelhano & Krzyś, 2020; Oliva & Torralba, 2007; Võ et al., 2019). On the basis of the knowledge that specific objects are likely to appear only in certain parts of a scene (e.g., an airplane in the sky, a car on the road), large parts of a scene can be excluded from the search space. Neuroimaging studies have shown that such global contextual expectations about likely target locations are reflected in the visual cortex. In one functional MRI (fMRI) study, participants searched for target objects in scenes (Preston et al., 2013). The scenes were selected such that the target would most likely be located on either the left or right side of the scene (e.g., searching for a bike in a scene with a bike path on the left). To isolate contextual expectations, the analyses focused on target-absent scenes. Results showed that multivariate activity patterns in the object-selective visual cortex (OSC; Fig. 1) carried information about the likely target location (left vs. right). Furthermore, this information correlated, across scene images, with the expected location of the target as rated by independent participants. These results indicate that spatial contextual expectations may be mediated by location-specific modulation of neural activity in the visual cortex.

**Fig. 2:**
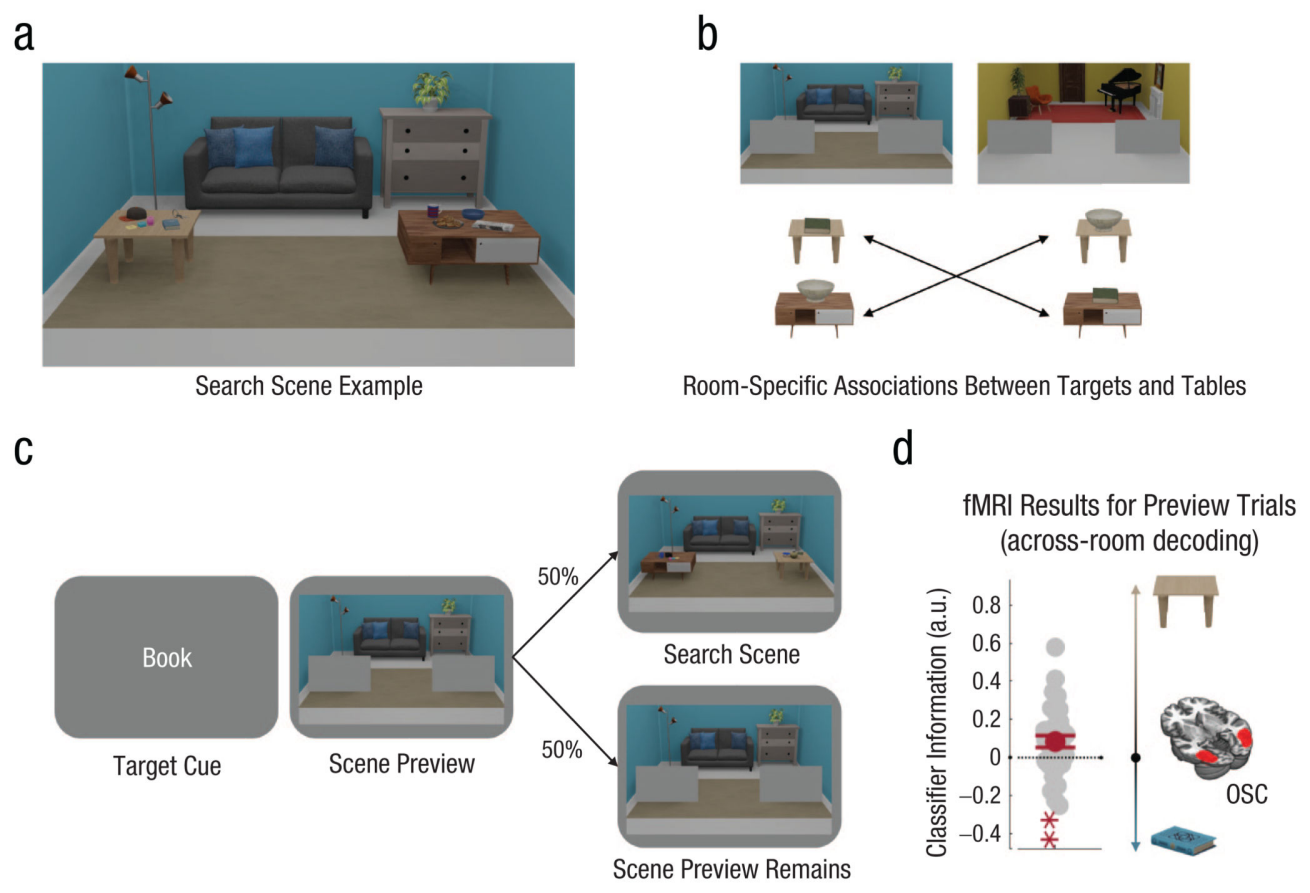
Design and results of an fMRI study investigating contextually guided search (Lerebourg et al., 2024). In (a) an example search scene, participants searched for targets (books and bowls) on top of tables in two rooms (recognizable by their blue or yellow walls). In (b) each room, each search target was associated with one of the two tables. The target-table association swapped across rooms. Each trial started with (c) a cue indicating the target, followed by a preview of the room, allowing participants to prepare for the search. On 50% of the trials, participants then performed a search task, indicating whether the target was present or absent. Crucially, on the other 50% of trials, the scene preview remained, and participants did not perform the search task—these trials were further analyzed. Multivariate activity patterns in the OSC were analyzed for the preview-only trials to isolate preparatory activity. Classifiers were trained to decode the target/table combination of one room (e.g., Book/Table 1 vs. Bowl/Table 2) and tested on the other room in which the associations were swapped (e.g., Bowl/Table 1 vs. Book/Table 2). Because of the swapping across rooms, (d) positive classifier information indicated that activity patterns reflected the table, whereas negative information indicated that activity patterns reflected the target. Results showed that preparatory activity in the OSC reflected the table rather than the target (i.e., it reflected the object that was most useful for guiding search). fMRI = functional MRI; OSC = object-selective visual cortex. **p < .01.

In addition to global scene layout, local object context also guides attention (Mack & Eckstein, 2011; for reviews, see Võ et al., 2019; Yu et al., 2023). For example, when searching for a toothbrush in a bathroom, it is efficient to first attend to the sink because it is closely associated with the toothbrush but is larger and therefore easier to find than the toothbrush itself. A recent fMRI study investigated the neural basis of this kind of search (Lerebourg et al., 2024). Participants searched for small objects that were contextually associated with tables (Fig. 2b). During the preparatory phase of the search, when the objects and tables were not yet visible, multivariate activity patterns in the OSC represented the table that was contextually associated with the target object rather than the target object itself (Fig. 2d). Together, these studies indicate that scene context guides spatial attention in two ways: by modulating visual cortex activity representing the parts of space in which target objects are likely to appear and by priming representations of objects that are contextually associated with the target.

### Context Shapes Preparatory Activity in the Visual Cortex

Scene context provides information not only about likely target locations but also about likely target appearance. For example, a target object far away in a scene will appear smaller than the same object nearby (although size-constancy mechanisms will partly reduce this perceptual difference). These contextual size constraints can be used, in principle, to inform the preparatory search template of the target (Peelen & Kastner, 2014). For example, when searching for a car in the distance, neurons tuned to small boxy shapes could be internally activated, resulting in the biased visual processing of the scene in favor of small boxy objects. In this case, size-mismatching targets may be missed, a prediction that has been confirmed by behavioral work (Eckstein et al., 2017).

**Fig. 3:**
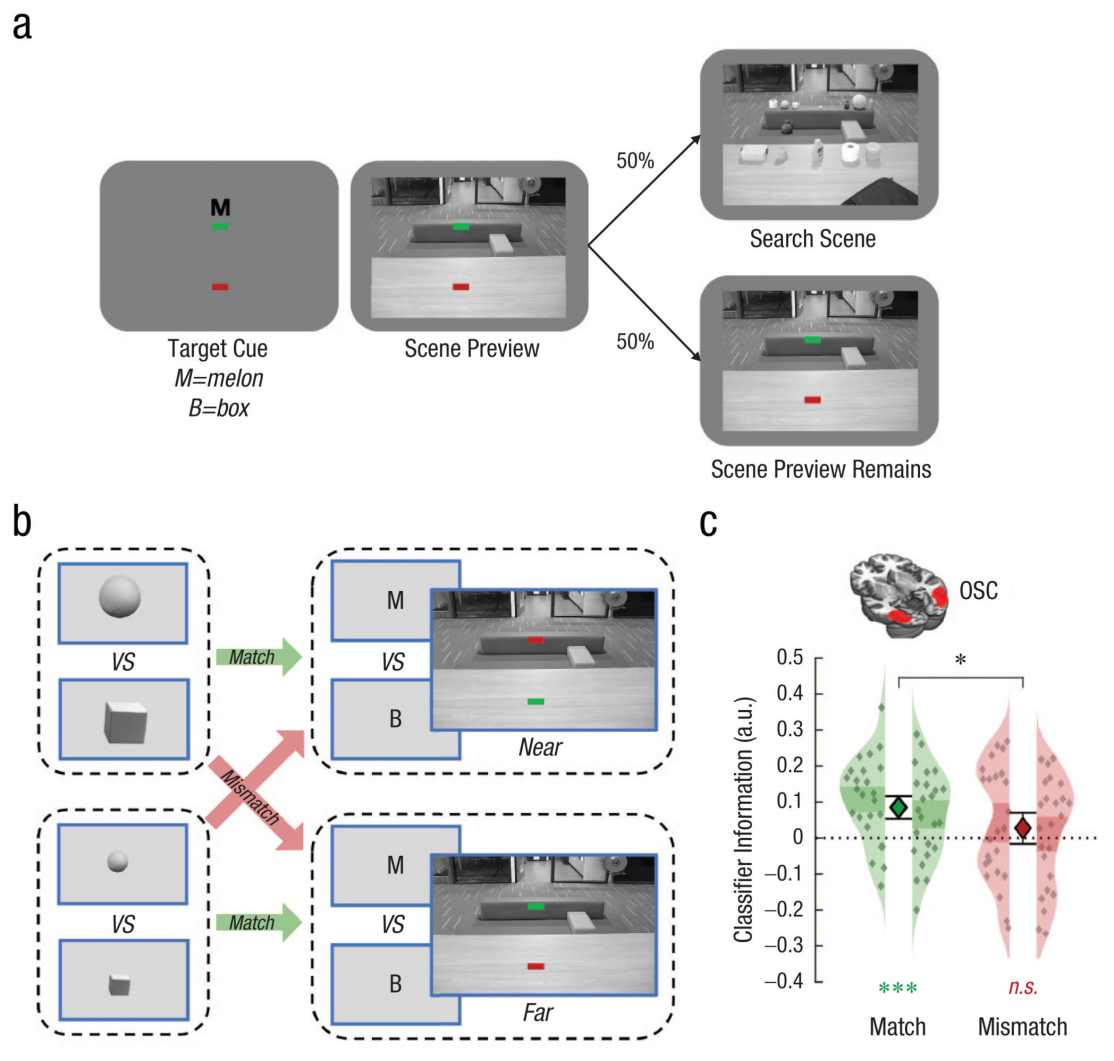
Design and results of an fMRI study investigating contextual influences on preparatory search activity in the OSC (Gayet & Peelen, 2022). On each trial, participants viewed (a) a cue indicating the target (melon or box) and the location of search (indicated by the green line). This was followed by a scene preview allowing participants to prepare to search nearby (1.5-m distance) or far away (3-m distance). On 50% of the trials, participants then performed a search task indicating whether the target was present or absent. Crucially, on the other 50% of trials, the scene preview remained, and participants did not perform the search task—these trials were further analyzed. Multivariate classifiers were trained on (b) activity evoked by isolated objects (melon vs. box) and tested on activity for the preview-only trials (M vs. B cue). The size of the objects used for classifier training could match (green arrows) or mismatch (red arrows) the size of the anticipated search target. Results in the OSC for the preview-only trials showed that (c) decoding of preparatory activity (classifier information, here shown for both hemispheres separately as well as averaged across hemipsheres) was significantly higher when the size of the training and testing set matched than when they mismatched, indicating that preparatory activity incorporated contextual expectations about target size. fMRI = functional MRI; OSC = object-selective visual cortex. *p < .05; ***p < .0005; n.s., not significant.

Neural evidence for the hypothesis that context shapes preparatory activity was provided by an fMRI study that investigated search for objects at different distances (Gayet & Peelen, 2022). Participants were cued to search for target objects either near or far in the scene (Fig. 3a). On a proportion of trials, participants prepared to search for the target at a particular distance, but the search array was omitted such that neural activity on these trials reflected top-down preparatory processes (Fig. 3b). Results showed that activity patterns in the OSC carried information about the shape of the cued target, resembling activity patterns evoked by the same object when visually presented in isolation. Importantly, these shape-specific activity patterns were modulated by scene context such that they best matched the corresponding shapes when visually presented at the contextually congruent retinal size (Fig. 3c). These results show that preparatory activity flexibly incorporates contextual expectations about target appearance. These contextually informed templates could then serve to quickly guide attention to possible target objects and enhance the representation of size-matching objects (Welbourne et al., 2021).

### Context Disambiguates Object Representations in the Visual Cortex

The studies in the previous sections investigated the neural basis of contextual influences on attentional guidance—the process that leads observers to spatially attend (or fixate) a candidate target object. In models of visual search, attentional guidance is followed by a verification stage in which the candidate target object is identified, and a decision is made about whether it matches the target (Eimer, 2014; Wolfe, 2021; Yu et al., 2023). Behavioral studies have shown that scene context facilitates this stage as well such that objects that are positioned in their original scene context are better and more quickly identified than objects that are positioned out of context (Biederman et al., 1982; for a review, see Bar, 2004).

**Fig. 4:**
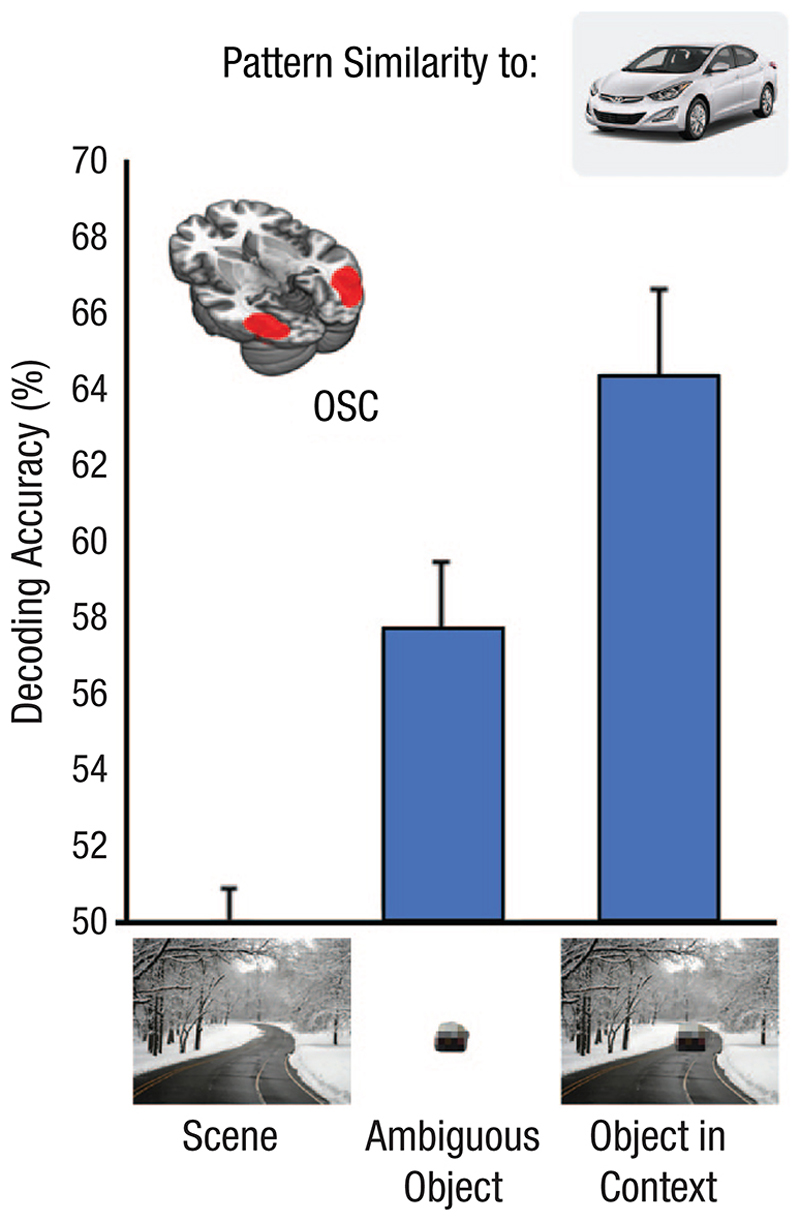
Results of an fMRI study investigating the facilitatory effect of scene context on object representations in the OSC (Brandman & Peelen, 2017).Multivariate classifiers were trained to distinguish clearly visible animate versus inanimate objects (e.g., the car shown in the inset) and tested on distinguishing ambiguous (degraded) objects presented alone or within scene context. Classification was significantly enhanced when the objects were presented in context, even though the context itself did not provide information about object category (scene-alone condition; leftmost bar). These results show that context facilitates object representations in the OSC, supporting the identification of these objects during visual search. fMRI = functional MRI; OSC = object-selective visual cortex.

To reveal the neural basis of the contextual facilitation of object identification, a series of neuroimaging studies presented degraded objects that were hard to recognize when presented in isolation but could still be recognized when presented in context (Fig. 4). Activity patterns evoked by the degraded objects (with and without scene context) were compared with activity patterns evoked by the same objects when presented centrally and in isolation, when they were clearly recognizable. Results from fMRI and magnetoencephalography (MEG) showed that context modulated neural response patterns in such a way that degraded object responses in the OSC became more similar to the corresponding intact object responses from around 300 ms after stimulus onset (Fig. 4; Brandman & Peelen, 2017). This contextual facilitation appears to be relatively automatic in that it does not require objects to be task-relevant (Leticevscaia et al., 2024), unlike the contextual influences on attentional guidance described in the previous sections. Finally, studies using transcranial magnetic stimulation (TMS) have shown that the OSC is causally linked to the facilitation of object identification based on global (scene) and local (object) context (Kim et al., 2011; Wischnewski & Peelen, 2021).

### Context Compression Through Object Grouping

Reaction times (RTs) in visual search tasks linearly scale with the number of distractor items, with the slope of the RT × set size function being a measure of visual search efficiency. For highly efficient (i.e., easy) searches (e.g., searching for a red item surrounded only by blue items), the slope is close to 0 ms/item such that the RT is minimally influenced by set size, whereas for more difficult searches, slopes of up to 50 ms/item are frequently observed (Wolfe et al., 2011). Surprisingly, behavioral studies have observed flat (or even negative) slopes for naturalistic visual search despite the scenes containing visually diverse objects (Neider & Zelinsky, 2008; Wolfe et al., 2011). These findings indicate that the concept of set size may not straightforwardly apply to natural scenes because it is hard to know what counts as an item in a naturalistic scene (e.g., whether each tree in a forest counts as a separate item). Indeed, objects in scenes can often be grouped on the basis of low-level (e.g., color) and mid-level (e.g., Gestalt principles) cues, thereby effectively reducing the set size (or clutter) of the scene.

Neuroimaging studies have shown that clutter can also be reduced by grouping objects on the basis of higher level positional regularities that have been learned over a lifetime (Baldassano et al., 2017; Kaiser & Peelen, 2018). In one study, two objects were presented in congruent or incongruent relative positions such that they either formed a meaningful constellation or not (e.g., a sofa in front of or behind a television; Kaiser and Peelen, 2018). Results showed that the congruent object displays evoked response patterns in the anterior OSC that were distinct from linear combinations of the individual object response patterns, pointing to an integrated representation of congruently positioned objects (Baldassano et al., 2017; Kaiser & Peelen, 2018). Another study showed that integrating representations of distractor objects reduces clutter such that targets are easier to find and are more strongly represented in the visual cortex (Kaiser et al., 2014). Together, these studies show that regularly arranged objects can be grouped in the visual cortex, thereby reducing the complexity of scene context and contributing to the efficiency of naturalistic visual search.

### Interactions Between the OSC and Other Brain Regions

Although the focus of the studies reviewed here is on the OSC, the reported modulations likely reflect top-down input from regions outside of the visual cortex. For example, in the studies on global and local spatial contextual expectations, similar (though weaker) results were reported in the intraparietal sulcus (IPS; Lerebourg et al., 2024; Preston et al., 2013). Therefore, the IPS may constitute the source of the biases observed in the OSC in these studies. When contextual expectations rely on recently learned associations (e.g., remembering that you left your keys on the table), memory systems, including the hippocampus, additionally modulate the visual cortex (Stokes et al., 2012; for a review, see Sherman & Turk-Browne, 2024). Finally, scene-selective regions may drive contextual expectations. For example, in the studies investigating the contextual facilitation of object representations, activity in the scene-selective cortex was correlated with the modulatory effects in the OSC (Brandman & Peelen, 2017) and was causally involved in the contextual facilitation of object-identification performance (Wischnewski & Peelen, 2021). Clearly, a full understanding of the neural basis of naturalistic visual search would require a better understanding of the interactions between the OSC and networks involved in attention, prediction, memory, and scene perception.

## Future Directions

By including scene context, the studies reviewed here have moved closer to real-world conditions than previous studies using artificial arrays. However, there are several remaining differences in how context affects search in daily life and in the laboratory. One important difference is that real-world search is situated in a broader temporal and spatial context; for example, we are usually already familiar with our environment before starting a search, and the scene will remain available throughout our search. Second, outside the laboratory, search is dynamic, with both objects and the observer potentially moving during the search. This creates the possibility for additional contextual expectations (e.g., about the typical trajectory of objects and their speed relative to the observer). Finally, search in daily life is typically internally driven rather than externally cued: We naturally look for objects or information that are needed for the task at hand (e.g., preparing a sandwich), and these searches themselves may be part of a structured (contextualized) sequence. These and other topics will be exciting avenues for future research.

## Conclusion

Neuroimaging studies have started to reveal various ways in which context is reflected in brain activity during visual search, particularly in the visual cortex. These studies have revealed that top-down attention and contextual expectations interactively drive preparatory activity and jointly modulate object processing. These studies bring us closer to understanding the neural basis of naturalistic visual search and point to directions for future research.
